# Digital Versus Conventional Rehabilitation After Total Hip Arthroplasty: A Single-Center, Parallel-Group Pilot Study

**DOI:** 10.2196/14523

**Published:** 2019-06-21

**Authors:** Fernando Dias Correia, André Nogueira, Ivo Magalhães, Joana Guimarães, Maria Moreira, Isabel Barradas, Maria Molinos, Laetitia Teixeira, Joaquim Pires, Rosmaninho Seabra, Jorge Lains, Virgílio Bento

**Affiliations:** 1 Neurology Department Hospital de Santo António-Centro Hospitalar do Porto Porto Portugal; 2 SWORD Health Porto Portugal; 3 Department of Population Studies Abel Salazar Institute of Biomedical Sciences Porto Portugal; 4 Centro de Investigação em Tecnologias e Serviços de Saúde Abel Salazar Institute of Biomedical Sciences University of Porto Porto Portugal; 5 EPIUnit Instituto de Saúde Pública Universidade do Porto Porto Portugal; 6 Orthopaedics Department Hospital da Prelada-Domingos Braga da Cruz Porto Portugal; 7 Physical Rehabilitation Medicine Department Rovisco Pais Medical and Rehabilitation Centre Tocha Portugal; 8 Engineering Department Instituto Universitário da Maia Maia Porto Portugal

**Keywords:** THA, THR, digital physiotherapy, telerehabilitation, biofeedback, motion trackers, AI-powered rehabilitation

## Abstract

**Background:**

The demand for total hip arthroplasty (THA) is rising. In the face of rapidly increasing health care costs, ensuring widespread, cost-effective rehabilitation is a priority. Technologies allowing independent home-based rehabilitation may be the key to facilitate access, improve effectiveness, and lower costs of care.

**Objective:**

The aim of this study was to assess the feasibility of a novel artificial intelligence–powered digital biofeedback system following THA and compare the clinical outcomes against supervised conventional rehabilitation.

**Methods:**

This was a single-center, parallel-group pilot study, with an 8-week intervention program. Patients were assessed at baseline, during the program (at 4 and 8 weeks), and 3 and 6 months after surgery. The primary outcome was the Timed Up and Go (TUG) score and secondary outcomes were the Hip dysfunction and Osteoarthritis Outcome Scale (HOOS; a patient-reported outcome) and hip range of motion (ROM).

**Results:**

A total of 66 patients were included: 35 digital physiotherapy (PT) versus 31 conventional. There were no differences at baseline between groups except for lower HOOS quality of life (QoL) subscale scores in the digital PT group. Clinically relevant improvements were noted in both groups at all time points. The digital PT group showed a retention rate of 86% (30/35). Per-protocol analysis revealed a superiority of the digital PT group for all outcome measures. Intention-to-treat analysis revealed the superiority of the digital PT group at all time points for TUG (change between baseline and 4 and 8 weeks: *P*<.001; change between baseline and 3 and 6 months: *P*=.001 and *P*=.005, respectively), with a difference between median changes of −4.79 seconds (95% CI −7.24 to −1.71) at 6 months post-THA. Between baseline and month 6, results were also superior in the digital PT group for the HOOS sports and QoL subscales and all ROM except for standing flexion.

**Conclusions:**

This study demonstrates this novel solution holds promise in rehabilitation after THA, ensuring better clinical outcomes than conventional rehabilitation while reducing dependence on human resources.

**Trial Registration:**

ClinicalTrials.gov NCT03045549; https://clinicaltrials.gov/ct2/show/NCT03045549

## Introduction

The demand for total hip arthroplasty (THA) is rising [1,2]. By 2030, primary THA in the United States is estimated to increase by 174% and revision THA by 137% compared to 2005 [2], to approximately 572,000 primary and 96,700 revision procedures per year [2].

The efficacy of THA is well documented [3-5], and rehabilitation is key to optimize outcomes [6,7]. Furthermore, studies indicate that more intensive and early progressive exercise leads to better outcomes [8,9], greater satisfaction and adherence [10,11], and reduction of complications and expenses [11,12]. In an expert consensus on best practices for rehabilitation after THA, the greatest support was for 4 to 8 weeks of therapeutic exercise, two to three times per week [13].

In the face of rapidly increasing health care costs, ensuring widespread cost-effective rehabilitation is a priority, but putting this into effect constitutes a challenge, both in terms of logistics and costs.

In recent years, telerehabilitation solutions (ie, rehabilitation services delivered at home from a remote location through a telecommunication system and information technology [14]) have been developed that allow professionals to remotely monitor rehabilitation programs [15-17]. These solutions have demonstrated a potential to reduce health care costs associated with supervision, facility provision, and transport of patients [18-21], while yielding similar, but not superior, clinical outcomes as conventional physical therapy post-THA [22,23].

Using a different approach, several authors have compared unsupervised home-based programs with physiotherapist-led outpatient rehabilitation programs, with both cases showing similar results for patients who comply with their program [21,24-26]. However, in studies comparing supervised with unsupervised training, or no recommended training at all, there is high variability in adherence rates, which is a well-accepted key determinant to therapy success [27-29], ranging from 23% to 85% [8,27,30,31].

More advanced technological solutions have emerged that incorporate biofeedback systems with the intent of increasing both patient performance and adherence [17,32,33] to maximize outcomes. Promising as these may be, they are generally poorly interactive and show low-level evidence, with no long-term validation studies available.

In a previous study, we tested a novel digital biofeedback system based on inertial motion trackers that enables independent home-based physical rehabilitation with remote monitoring from a clinical team after total knee arthroplasty (TKA) [34]. In this study (N=59; NCT03047252), we compared the digital system to conventional, face-to-face, home-based rehabilitation post-TKA over an 8-week program. The results demonstrated that this solution was safe and very well-accepted, with high adherence and satisfaction levels and, most importantly, that the clinical outcomes were superior to conventional rehabilitation [34]. These encouraging results prompted further studies, with the intent of validating this solution in other therapeutic scenarios.

The aim of this single-center, parallel-group pilot study is to assess patient uptake and system safety in patients undergoing THA, as well as to compare the clinical outcomes of a home-based program using this digital physiotherapy (PT) system against conventional, in-person, home-based rehabilitation after THA.

## Methods

### Study Design

This was a single-center, parallel-group pilot study. It was designed to assess patient uptake and safety of a digital physiotherapy system, as well as to compare the clinical outcomes of a home-based program using a home-based digital program compared with conventional, in-person, home-based rehabilitation after THA.

### Study Timeline

All consecutive patients admitted for THA between December 19, 2016 and January 16, 2018, were screened preoperatively and postoperatively for eligibility at Hospital da Prelada, Porto, Portugal, by the two orthopedic surgeons that oversaw the study (JP and RS). Completion date for the 6-month follow-up assessment was July 16, 2018.

### Inclusion and Exclusion Criteria

All patients included in this study were referred to post-THA rehabilitation by two independent physicians. Patients were included if they were (1) aged 18 years or older and had (2) clinical and imaging (CT) evidence of hip osteoarthritis as assessed by the orthopedic surgeon, (3) indication for THA according to the patient´s orthopedic surgeon, (4) ability to walk (unaided or with assistive device), and (5) availability of a caregiver to assist the patient after surgery.

Exclusion criteria were (1) admitted for revision THA; (2) contralateral hip or knee osteoarthritis severely limiting patient mobility and ability to comply with a rehabilitation program; (3) aphasia, dementia, or psychiatric comorbidity interfering with communication or adherence to the rehabilitation process; (4) respiratory, cardiac, metabolic, or other condition incompatible with at least 30 minutes of light to moderate physical activity; (5) major medical complications occurring after surgery that prevented the discharge of the patient within 10 days after the surgery; (6) other medical or surgical complications that prevent the patient from complying with a rehabilitation program; and (7) blindness or illiteracy.

### Patient Allocation

Patients were recruited at Hospital da Prelada, Porto, Portugal. Patient allocation was performed using patient address as the criterion. Those patients residing in areas outside the administrative limits of the city of Oporto were allocated to the digital PT group, whereas those residing within the city limits were allocated to the conventional rehabilitation group. Patient allocation was performed centrally by one investigator (FDC) and communicated to the responsible physiotherapist only after patient enrollment.

### Blinding

The nature of the study did not allow blinding of the patients. Patient assessment was performed by two investigators (JP and RS), who were blinded to the study groups. Statistical analysis was performed by a blinded statistician (LT).

### Intervention

After the initial assessment, all patients were submitted to elective THA. Surgical technique was the same for all patients—direct lateral approach under regional anesthesia.

Between day 1 postop and hospital discharge, all patients were taught how to safely get in and out of bed and were asked to perform alternate ankle flexion and extension exercises regularly. All patients performed initial gait training with canes.

After hospital discharge, both groups received an 8-week rehabilitation program starting between day 7 and day 10 after surgery (see Multimedia Appendix 1). These were designed based on the results of a Delphi panel on best practices for rehabilitation after THA [13] and the protocols published by SOFMER, the French Physical and Rehabilitation Medicine Society [35].

In the digital PT group, patients received an initial visit from the physical therapist to assess specific needs and to teach patients and caregivers how to set up and use the system. Patients then performed exercise sessions independently, using the system, under asynchronous remote monitoring from the physical therapist (see Multimedia Appendix 1 for more details). Patients were instructed to exercise 5 to 7 days per week, minimum 30-minute sessions, but they were not excluded in case of lower adherence. Each patient received a telephone call on weeks 2 and 6 to check on patient adaptation, review the program, and assess adverse events; a face-to-face visit on week 4 to perform an in-depth review of the program; and a termination visit to collect the system. Additional visits were performed when required.

The conventional rehabilitation group received a home-based supervised program provided by a physiotherapist, three times a week, for 1 hour (see Multimedia Appendix 1 for more details). Patients were also instructed to perform additional sessions on at least two other days of the week. These were nonmandatory, and no record of these sessions was kept.

### Outcomes Assessment

#### Total Therapist Time

Total therapist time was calculated in both groups, considering the time spent on face-to-face contacts and spent in travel and on calls. For the digital intervention group, time spent per patient in the Web-based portal was also calculated.

#### Safety and Adverse Events

In the digital PT group, patients were asked to rate pain and fatigue on a scale from zero to 10 at the end of each session. These were available for remote monitoring through the portal. Patients were also given the direct contact of the assigned physical therapist to report adverse events: pain during exercise, falls, and other medical complications (eg, inflammatory signs or infection on the surgical wound or operated member; thrombophlebitis).

Patients in the conventional rehabilitation group performed supervised sessions by a physical therapist, enabling early adverse event detection and reporting.

#### Primary and Secondary Outcomes

For primary outcome, we chose a performance test—the Timed Up and Go (TUG) test [36], which measures patient mobility and consists of the time it takes to rise from a chair, walk 3 meters, turn around, walk back to the chair, and sit down. This test is among the most recommended outcome measures to routinely assess or monitor outcomes after primary THA [13]. It is simple, practical, and quick and easy to administer, plus it has been demonstrated to predict both short- [37] and long-term [38] function following hip arthroplasty. Importantly, it has also shown excellent interrater (intraclass correlation [ICC] ≥0.9) and very good test-retest (ICC 0.8-0.89) reliability in patients with elective hip replacement (N=100) [39], and higher sensitivity to change in performance after THA than other commonly used self-reported measures, such as the Western Ontario and McMaster Universities Osteoarthritis Index (WOMAC) and the Lower Extremity Functional Scale (LEFS) [40]. Moreover, Podsiadlo and Richardson [36] confirmed its content validity in elderly persons (N=60), in that it evaluated a well-recognized series of maneuvers used in daily life.

Secondary outcomes were (1) patient-reported outcomes, measured by the Hip dysfunction and Osteoarthritis Outcome Scale (HOOS) [41] and (2) hip range of motion (ROM).

The HOOS consists of five subscales: (1) pain, (2) symptoms, (3) function in activities of daily living (ADL), (4) function in sport and recreation (sport), and (5) hip-related quality of life (QoL). Patients are asked to answer this disease-specific questionnaire, based on the previous week, with standardized options for each question (each is assigned a score from 0-4). A normalized score (100 indicating no symptoms and 0 indicating extreme symptoms) is calculated for each subscale. This scale has shown high test-retest reproducibility for people with hip disability with or without hip osteoarthritis, with ICC ranging from 0.75 to 0.97 for all subscales [41]. The HOOS content validity was tested by Nilsdotter and colleagues [42] in patients assigned to THA (n=90), by asking them to rate the importance of each item. All items were considered to be of at least some importance by more than 67% of the patients, the limit set to justify inclusion into the HOOS. All items included in the pain (10/10), ADL (17/17), sport (5/5), QoL (4/4), and most items included in symptoms (4/5), were considered at least somewhat important by more than 80% of patients.

The SWORD device was used in both groups to measure active hip ROM. This device has been certified for use as an angle-measurement tool, with a reported root mean square error of 3.5° compared with standard goniometry in the technical file. Active hip ROM was measured in degrees in the following exercises: lying and standing hip flexion, lying and standing hip abduction, and standing hip hyperextension. For each exercise, the patient was asked to perform three repetitions by itself; the best value of the three was recorded.

Patients were assessed at baseline (preoperatively), 4 weeks after initiation of rehabilitation, at the end of the 8-week program, and at 3- and 6-months follow-up evaluations.

### Sample Size Estimation

Calculations were performed taking into consideration the primary outcome measure—TUG—and based on a minimal detectable change of 2.49 seconds, as reported by Kennedy et al [43] on a longitudinal study evaluating outcomes following total hip and knee arthroplasty. Considering an effect size of 0.65, a power of 80%, and a two-sided .05 significance level, 60 patients (30 in each group) would be necessary to detect a difference of 2.49 seconds between the two groups. Considering a dropout rate of 15%, the target recruitment was 70 patients.

### Statistical Analysis

To assess differences in clinical and demographic variables of the patients allocated to the two study groups, independent samples *t* test or Mann-Whitney *U* test were used for quantitative variables. For categorical variables, chi-square test or Fisher exact test were used.

Outcome analysis was performed using both an intention-to-treat analysis and a per-protocol analysis. Differences between interventions were evaluated using independent samples *t* test or Mann-Whitney *U* test. For nonnormally distributed variables, the magnitude of the difference in the medians was assessed using Hodges-Lehman estimator. Additionally, a repeated measures ANOVA was also performed, with group as an independent factor and time as a within-subjects factor. When necessary, logarithm transformation was performed to obtain normally distributed variables. In all analysis, a significance level of .05 was considered. Statistical analysis was performed using IBM SPSS version 24.0.

### System Technical Specifications

**Figure 1:**
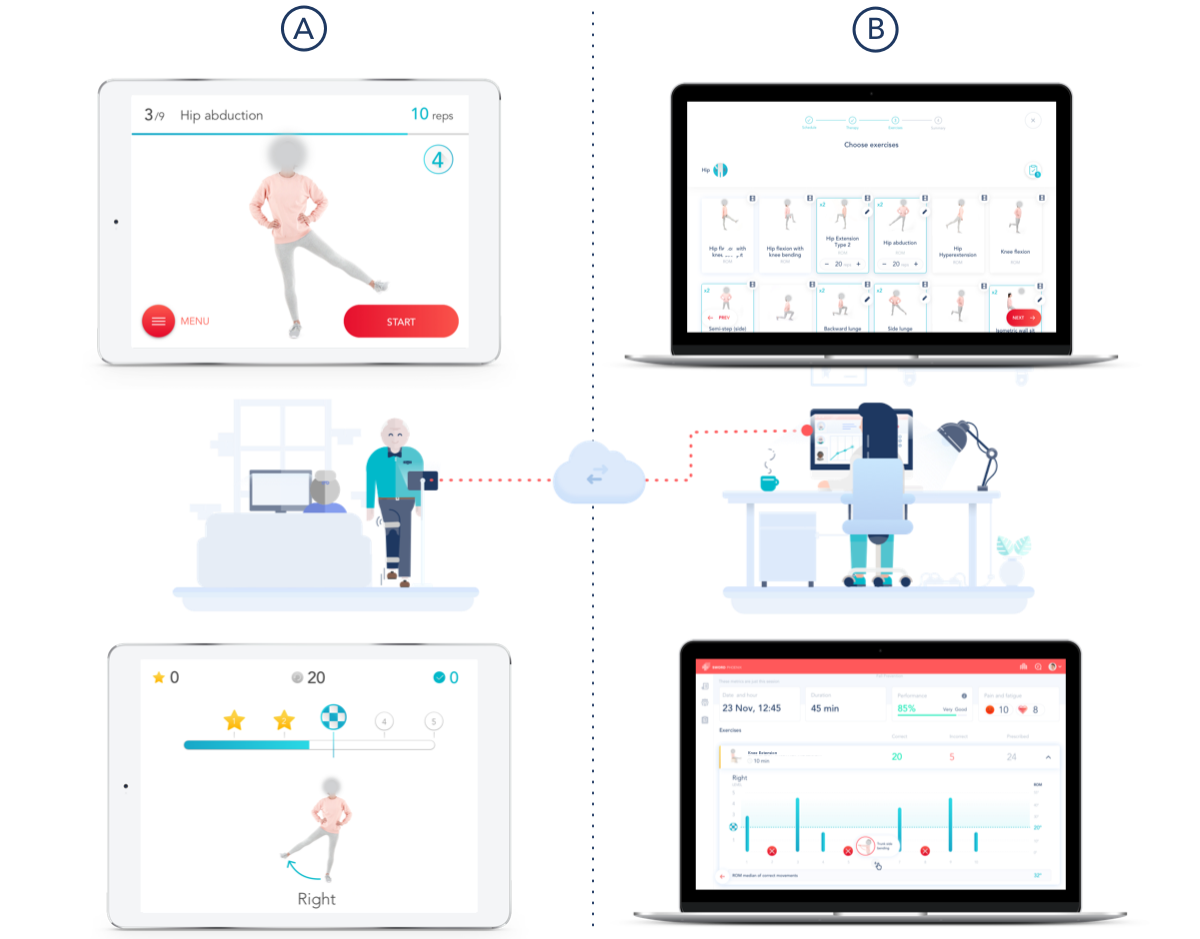
System components. (A) Mobile app. Preparation screen (top left): this screen displays video and audio instructions for each exercise. Execution screen (bottom left). (B) Web portal. Prescription screen (top right) displaying the exercise list and session layout. Results screen (bottom right) presenting (1) date, time, and session duration; (2) pain and fatigue scores; and (3) information on each repetition-range of motion and movement errors.

The system consisted of the elements described subsequently (Figure 1).

#### Inertial Motion Trackers

Each tracker consisted of a gyroscope, an accelerometer, and a magnetometer, which enabled precise movement quantification. The trackers were placed on body segments using Velcro straps in three specific positions: (1) over the sternal manubrium (red tracker), (2) on the anterior surface of the hip (green tracker), and (3) over the anterior tibial crest (blue tracker).

#### Mobile App

The app guided the patient through the session, providing video and audio instructions before each exercise, as well as real-time audio and video biofeedback during the exercise. If the patient performed a movement error or assumed an incorrect posture, an error message was displayed, allowing the patient to correct the movement in the following attempts.

#### Web-Based Portal

The portal enabled remote result monitoring and exercise prescription/edition by the clinical teams.

### Ethics Approval of Research

The study was approved by the National Data Protection Commission (authorization number 1476/2017) and by the local ethics committee at Hospital da Prelada (Chair: Dr Juiz Conselheiro Almeida Lopes). The methods were conducted in accordance with the approved guidelines. All patients and caregivers were provided with information about the purpose and procedures of the study and provided written informed consent before inclusion. All patient data were anonymized and linked to the patient by a unique study number that did not contain any personal identifiers.

### Data Availability

Individual participant data that underlie the results reported in this article will be shared after deidentification as supplementary information (Multimedia Appendix 2) of this paper. Other documents, namely the study protocol, Consolidated Standards of Reporting Trials (CONSORT) details, will also be made permanently available immediately following publication, either through the online version of this paper or at ClinicalTrials.gov (UI: NCT03045549).

## Results

### Overview

**Figure 2:**
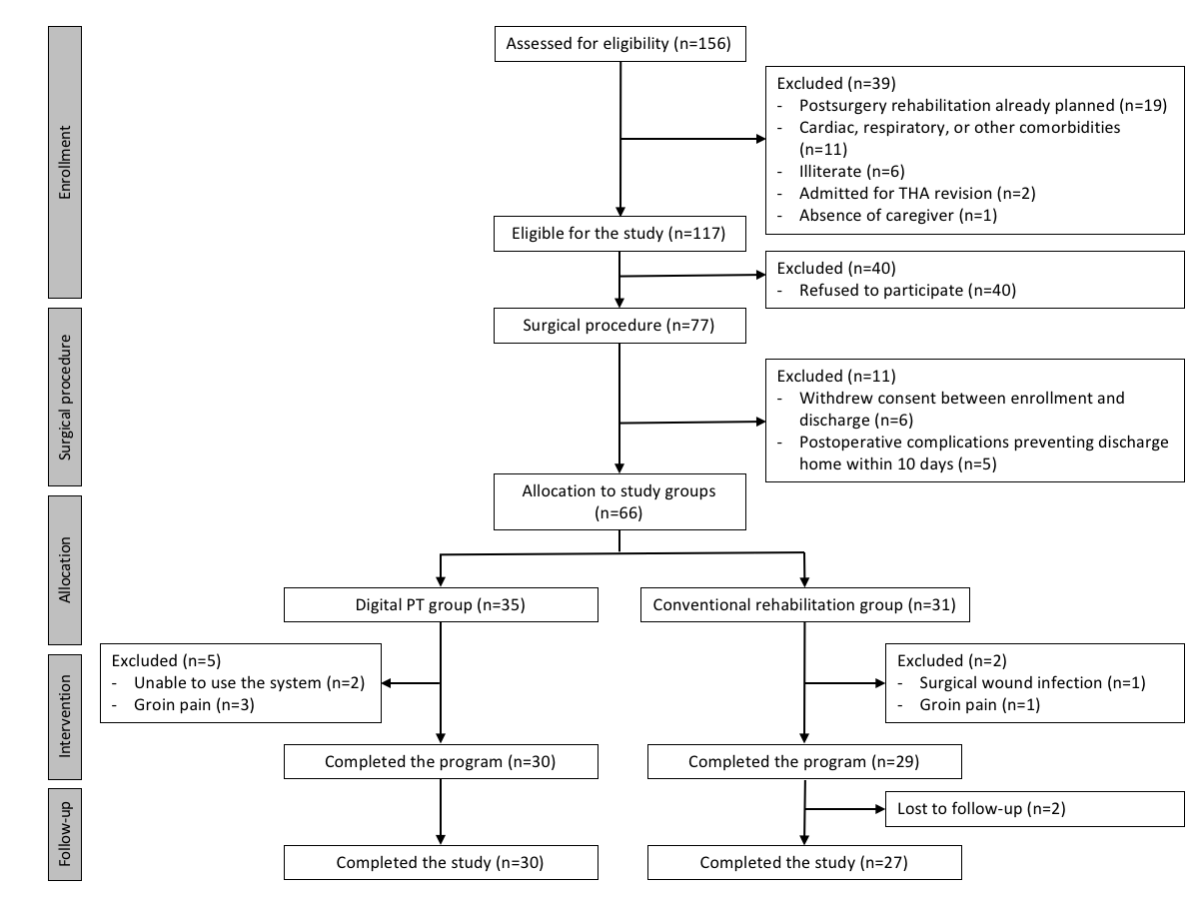
CONSORT (Consolidated Standards of Reporting Trials) diagram. PT: physiotherapy; THA: total hip arthroplasty.

A total of 156 patients were assessed for eligibility between December 19, 2016 and January 16, 2018. Figure 2 shows the CONSORT diagram for the study (see also Multimedia Appendix 3). The study inclusion rate was of 42% (66/156). Between initial assessment and patient allocation, 90 patients refused to participate or withdrew consent, corresponding to 58% of all screening failures.

Overall, 66 patients were included (35 in the digital PT group versus 31 in conventional rehabilitation). The dropout rate in the digital PT group was 14% (5/35): two patients did not adapt to the system and withdrew consent in the first week and three were excluded due to groin pain. The dropout rate in the conventional rehabilitation group was 6% (2/31): two patients were excluded, one due to a surgical wound infection requiring readmission and another due to groin pain. In total, 59 patients completed the study (30 versus 29) and 57 completed the follow-up assessments—two patients in the conventional rehabilitation group were lost to follow-up between the 3- and 6-month assessments.

### Study Population Characteristics

Baseline characteristics of study participants regarding demographics, comorbidities, and risk factors for adverse events, as well as data on hospitalization and surgery are summarized in Table 1 (divided by allocation group). There were no differences at baseline between the two study groups regarding any population characteristics.

### Independence of Use

In the digital PT group, 13 of 35 patients (37%) required the assistance of a caregiver for tracker or strap placement or navigation. Patients requiring assistance were older (mean age 68.0, SD 7.6 years versus mean 57.7, SD 6.6; *P*=.001).

**Table 1:** Baseline characteristics of study participants (N=66).

Population characteristics		Digital physiotherapy group (n=35)	Conventional rehabilitation (n=31)	*P* value
**Demographics**				
	Age (years), mean (SD)	62.4 (8)	66.6 (10)	.07^a^
	Gender (female), n (%)	15 (43)	16 (2)	.64
	Operated hip side (right), n (%)	16 (46)	12 (39)	.74
**Comorbidities and known risk factors for adverse events**				
	Body mass index, mean (SD)	28.3 (3)	27.4 (4)	.31^a^
	Smoking, n (%)	2 (6)	7 (23)	.07^b^
	Hypertension, n (%)	14 (40)	12 (39)	>.99
	Diabetes, n (%)	11 (31)	7 (23)	.59
	Pulmonary disease, n (%)	1 (3)	1 (3)	>.99
	Cardiac disease, n (%)	3 (9)	5 (16)	.46^b^
	Stroke, n (%)	1 (3)	0.0	—^c^
	Renal disease, n (%)	0.0	0.0	—
	Bleeding disorders, n (%)	0.0	2 (6)	—
	ASA^d^ (class 3 or 4), n (%)	8 (23)	10 (32)	.56
	Steroids for chronic condition, n (%)	0	0	—
	Previous contralateral hip replacement, n (%)	7 (20)	5 (16)	.93
	Previous knee replacement, n (%)	1 (3)	0	—
**Hospital admission and surgical procedure**				
	Time between admission and surgery (hours)	<24	<24	—
	Operative time (min), mean (SD)	63.7 (19)	59.9 (9)	.10^a^
	Noncemented prosthesis, n (%)	2 (6)	2 (6)	>.99
	Minor adverse events before discharge, n (%)	0.0	0.0	—
	Length of stay (days), median (IQR^e^)	6.0 (2)	6.0 (1)	.43^f^

^a^Independent sample *t* test.

^b^Fisher exact test.

^c^Not applicable.

^d^American Society of Anesthesiology physical status classification system.

^e^IQR: interquartile range.

^f^Mann-Whitney *U* test.

### Adherence to the Intervention

Only five patients (17%) did not comply with the recommended session frequency of five times per week.

### Patient Satisfaction

Patients in the digital PT group were asked to report their satisfaction level by answering the question: “On a scale from 0-10 (‘0’ meaning that you would not recommend and ‘10’ that you would highly recommend), how much would you recommend the system to one of your friends or neighbors?” Of the 35 patients in this group, 32 (91%) rated the system as 10, two patients rated the system as 9, and one did not answer.

### Therapist-Patient Interaction

Patients in the conventional rehabilitation group had 24 in-person sessions, whereas patients in the digital PT group had 3 face-to-face contacts with the therapist and, on average, 0.6 (range 0-2) extra contacts for technical assistance. Regarding telephone calls, in addition to the two scheduled calls per protocol, each patient received a median of four extra calls (range 0-7), the vast majority due to difficulties in interacting with the system.

### Treatment Intensity

Total active treatment time was similar in both groups in both intention-to-treat (ITT) and per-protocol analysis (ITT: *P*=.11; per protocol: *P*=.24). In the ITT analysis, treatment intensity in the digital PT group was 20 hours (interquartile range [IQR] 11.0, range 1.0-59.0) and in the per-protocol analysis was 21 hours (IQR 10.3, range 8.0-59) versus 24 hours in the conventional PT group.

### Outcomes Assessment

#### Total Therapist Time

Total therapist time was lower in the digital intervention group (mean 6.5, IQR 1.2 hours versus mean 32.1, IQR 5.2 hours; *P*<.001).

#### Safety and Adverse Events

For all patients enrolled in the study (66 patients), there was no significant difference between groups for safety and adverse events (*P*>.99).

In the digital PT group, the adverse event rate was 14% (5/35). Three patients were excluded due to significant pain during hip abduction, without inflammatory or other warning signs. All three patients recovered spontaneously within 2 weeks. One patient reported inflammatory signs over the surgical wound and another suffered a fall (not during system use), with no need for hospital assistance.

In the conventional rehabilitation group, the adverse event rate was 23% (7/31). One patient required hospital readmission and a revision procedure due to a surgical wound infection, one was excluded due to groin pain, two patients reported inflammatory signs over the surgical wound, one patient had a thrombophlebitis, one reported a unilateral lower limb edema (with spontaneous recovery), and one patient suffered a fall, with no need for hospital assistance.

#### Primary and Secondary Outcomes

##### Baseline

There were no differences between the two groups regarding outcome measures, except for the HOOS QoL subscale (*P*=.03; see Tables 2-4). The median difference between the TUG scores in the two groups was of 2.34 seconds (95% CI −0.69 to 5.17) in favor of the conventional rehabilitation group. Taking into consideration the 2.49 seconds reported as minimal detectable change for this test [43], this difference is neither statistically nor clinically significant.

**Table 2:** Primary outcome assessment of Timed Up and Go (TUG) test: intention-to-treat analysis (N=66).

Time point		TUG time (seconds), median (IQR^a^)		*P* value^b^	Estimate difference between groups (95% CI)
		Digital PT^c^ group (n=35)	Control group (n=31)		
Baseline		17.50 (6.33)	14.89 (9.42)	.12	2.34 (−0.69, 5.17)
**Short term**					
	8 weeks	7.26 (2.15)	11.03 (6.84)	<.001	−3.34 (−5.14, −1.70)
	Change baseline-8 weeks	−10.50 (7.45)	−2.90 (7.10)	<.001	−6.33 (−8.79, −3.42)
**Medium term**					
	6 months	6.38 (2.30)	8.20 (4.22)	<.001	−1.87 (−3.02, −0.62)
	Change baseline-6 months	−10.50 (7.39)	−5.10 (6.94)	.005	−4.79 (−7.24, −1.71)

^a^IQR: interquartile range.

^b^Mann-Whitney *U* test.

^c^PT: physiotherapy.

**Table 3:** Secondary outcome of patient-reported Hip dysfunction and Osteoarthritis Outcome Scale (HOOS): intention-to-treat analysis (N=66).

Time point and variable		Score, median (IQR^a^)		*P* value^b^	Estimate difference between groups (95% CI)
		Digital PT^c^ group (n=35)	Control group (n=31)		
**Baseline**					
	Symptoms	35.0 (20.0)	40.0 (30.0)	.12	−10.0 (−20.0, 0.0)
	Pain	33.0 (13.0)	33.0 (35.0)	.50	−3.0 (−13.0, 5.0)
	Activities of daily living	29.0 (15.0)	28.0 (28.0)	.75	1.0 (−6.0, 7.0)
	Sports	0.0 (6.0)	0.0 (19.0)	.34	0.0 (0.0, 0.0)
	Quality of life	13.0 (13.0)	19.0 (25.0)	.03	−6.0 (−13.0, 0.0)
**8 weeks**					
	Symptoms	100.0 (5.0)	95.0 (20.0)	.01	5.00 (0.0, 10.0)
	Pain	100.0 (7.0)	98.0 (12.0)	.24	0.0 (0.0, 5.0)
	Activities of daily living	93.0 (11.0)	82.0 (14.0)	<.001	9.0 (4.0, 13.0)
	Sports	50.0 (18.0)	38.0 (19.0)	.004	12.0 (6.0, 19.0)
	Quality of life	81.0 (19.0)	69.0 (31.0)	.08	6.0 (0.0, 18.0)
**Change baseline-8 weeks**					
	Symptoms	60.0 (30.0)	45.0 (30.0)	.06	10.0 (0.0, 20.0)
	Pain	60.0 (22.0)	60.0 (32.0)	.75	2.0 (−10.0, 10.0)
	Activities of daily living	56.0 (23.0)	57.0 (27.0)	.63	−2.0 (−10.0, 6.0)
	Sports	44.0 (25.0)	38.0 (25.0)	.26	6.0 (−6.0, 13.0)
	Quality of life	63.0 (31.0)	50.0 (25.0)	.46	6.0 (−6.0, 13.0)
**6 months**					
	Symptoms	100.0 (5.0)	95.0 (10.0)	.20	0.0 (0.0, 5.0)
	Pain	100.0 (5.0)	100.0 (7.0)	.75	0.0 (0.0, 0.0)
	Activities of daily living	96.0 (11.0)	88.0 (19.0)	.02	4.0 (0.0, 10.0)
	Sports	75.0 (32.0)	50.0 (32.0)	.01	19.0 (6.0, 37.0)
	Quality of life	94.0 (12.0)	81.0 (19.0)	.02	7.0 (0.0, 19.0)
**Change baseline-6 months**					
	Symptoms	60.0 (25.0)	45.0 (30.0)	.06	10.0 (0.0, 20.0)
	Pain	65.0 (18.0)	53.0 (30.0)	.21	7.0 (−5.0, 17.0)
	Activities of daily living	63.0 (22.0)	56.0 (25.0)	.10	7.0 (−1.0, 15.0)
	Sports	69.0 (31.0)	38.0 (38.0)	.004	25.0 (7.0, 37.0)
	Quality of life	75.0 (32.0)	56.0 (31.0)	.01	19.0 (6.0, 25.0)

^a^IQR: interquartile range.

^b^Mann-Whitney *U* test.

^c^PT: physiotherapy.

**Table 4:** Secondary outcome of hip range of motion assessment: intention-to-treat analysis (N=66).

Time point and variable		Median (IQR^a^)		*P* value^b^	Estimate difference between groups (95% CI)
		Digital PT^c^ group (n=35)	Control group (n=31)		
**Baseline**					
	Lying flexion	28.2 (19.1)	37.1 (20.0)	.07	−8.9 (−18.53, 0.67)
	Lying abduction	12.2 (5.4)	15.9 (9.1)	.05	−3.7 (−7.48, 0.02)
	Standing flexion	45.1 (15.9)	49.6 (16.7)	.27	−4.5 (−12.52, 3.53)
	Standing hyperextension	−11.9 (7.0)	−15.4 (8.8)	.31	3.4 (−0.44, 7.33)
	Standing abduction	23.5 (6.8)	25.8 (10.7)	.08	−2.2 (−6.78, 2.26)
**8 weeks**					
	Lying flexion	84.0 (23.5)	66.6 (19.6)	.002	17.5 (6.78, 28.18)
	Lying abduction	50.5 (17.5)	39.2 (15.2)	.01	11.4 (3.27:19.50)
	Standing flexion	87.6 (21.2)	80.0 (19.8)	.14	7.5 (−2.58, 17.66)
	Standing hyperextension	−36.7 (14.3)	−30.1 (8.2)	.03	−6.6 (−12.28, −0.96)
	Standing abduction	52.2 (13.8)	40.3 (11.3)	<.001	11.9 (5.62, 18.13)
**Change baseline-8 weeks**					
	Lying flexion	55.8 (27.4)	29.4 (25.6)	<.001	26.4 (13.32, 39.50)
	Lying abduction	38.4 (17.3)	23.3 (15.7)	<.001	15.1 (6.91, 23.25)
	Standing flexion	42.5 (21.3)	30.4 (20.3)	.02	12.0 (1.81, 22.33)
	Standing hyperextension	−24.7 (12.7)	−14.7 (10.1)	.001	−10.1 (−15.75, −4.38)
	Standing abduction	28.7 (13.4)	14.6 (13.5)	<.001	14.1 (7.51, 20.76)
**6 months**					
	Lying flexion	80.7 (24.4)	70.0 (19.3)	.06	10.7 (−0.27, 21.6)
	Lying abduction	49.8 (18.2)	41.6 (14.3)	.048	8.2 (0.06, 16.31)
	Standing flexion	90.2 (23. 1)	84.8 (19.8)	.32	5.4 (−5.25, 16.03)
	Standing hyperextension	−34.1 (15.1)	−28.8 (9.2)	.10	−5.3 (−11.36, 0.81)
	Standing abduction	51.7 (15.1)	43.8 (11.8)	.02	8.0 (1.24, 14.69)
**Change baseline-6 months**					
	Lying flexion	52.5 (26.6)	32.8 (25.6)	.003	19.6 (6.73, 32.50)
	Lying abduction	37.6 (18.2)	25.7 (15.2)	.01	11.9 (3.57, 20.20)
	Standing flexion	45.1 (22.6)	35.2 (20.6)	.07	9.9 (−0.79, 20.57)
	Standing hyperextension	−22.2 (13.3)	−13.5 (11.1)	.01	−8.7 (−14.72, −2.59)
	Standing abduction	28.2 (14.3)	18.0 (12.1)	.003	10.2 (3.64, 16.74)

^a^IQR: interquartile range.

^b^Mann-Whitney *U* test.

^c^PT: physiotherapy.

##### Short-Term Outcomes Assessment

###### 4-Week Assessment

Differences between groups were found for TUG between the digital PT and the conventional group: mean 9.9 (SD 5.4) seconds versus mean 15.0 (SD 8.2) seconds, respectively (*P*<.001), (see Multimedia Appendix 4) and for all hip ROM exercises, except standing flexion (*P*=.05; see Multimedia Appendix 4). There were no differences between groups in terms of patient-reported outcomes (see Multimedia Appendix 4).

###### 8-Week Assessment

The TUG scores were again lower in the digital PT group (*P*<.001; see Table 2). The median difference between the TUG scores in the two groups was 3.34 seconds (95% CI −5.14 to −1.70).

Regarding HOOS, the median scores in the digital PT group were superior to the conventional rehabilitation group for all subscales, except for pain and QoL (see Table 3). Importantly, in the symptoms and pain subscales, the median scores at the 8-week assessment were either the maximum score that can be attained (100) or close to that value in both groups, revealing a ceiling effect, which persisted over time (see Table 3).

Hip ROM was also higher in the digital PT group for all exercises, except for standing flexion (see Table 3).

###### Change Between Baseline and the 8-Week Assessment

The median difference between the changes in the two groups regarding the TUG score was 6.33 seconds (95% CI −8.79 to −3.42). The minimal detectable change was 2.49 seconds, which reveals a clinically significant difference (see Table 2).

No significant differences were detected in the median changes from baseline and week 8 for HOOS scores (see Table 3).

For hip ROM, significant improvements from baseline were noted in both groups, again with the digital PT group showing greater results (see Table 4).

In the per-protocol analysis, the change between baseline and week 8 was superior in the digital PT group for all outcome measures (see Multimedia Appendix 5).

##### Medium-Term Outcomes Assessment

###### 3-Months Assessment

The TUG score remained significantly different between groups (*P*<.001), with patients from the SWORD group experiencing better results (see Multimedia Appendix 4).

For the HOOS, the median scores in the digital PT group were superior for all subscales except for pain (*P*=.10) and symptoms (*P*=.08; see Multimedia Appendix 4).

Hip ROM was also higher in the digital PT group for all measured exercises (*P*<.001), except for standing flexion (*P*=.41; see Multimedia Appendix 4).

###### 6-Months Assessment

The median difference between the TUG scores in the two groups was 1.87 seconds (95% CI −3.02 to −0.62) in favor of the digital PT group (*P*=.002; see Table 2).

For HOOS, the median scores in the digital PT group were significantly superior to the conventional rehabilitation group for the ADL (*P*=.02), sports (*P*=.01), and QoL (*P*=.02) subscales (see Table 3). Importantly, the majority of patients from both groups reported the highest possible scores in the symptoms and pain subscales, and the ADL and QoL scores from the digital PT group nearly reached this same plateau (see Table 3).

Hip ROM was higher in the digital PT group for lying abduction (*P*=.048) and standing abduction (*P*=.02; see Table 4).

###### Change Between Baseline and the 6-Months Assessment

The ITT analysis revealed the superiority of the digital PT group in the TUG test, HOOS sports and QoL subscales, and all hip ROM exercises, except for standing flexion.

The median difference between the changes in the two groups for TUG was 4.79 seconds (95% CI −7.24 to −1.70) in favor of the digital PT group (see Table 2).

For HOOS, the difference between median score changes was both statistically and clinically significant in the sports (25.0 points, 95% CI 7.0-37.0) and the QoL (19.0 points, 95% CI 6.0-25.0) subscales (see Table 3).

For hip ROM, significant differences between the mean changes in the two groups were detected in all ROM exercises, except the standing flexion hip ROM (*P*=.07; see Table 4).

In the per-protocol analysis, the superiority of the digital PT group was verified for all outcome measures (see Multimedia Appendix 5).

#### Repeated Measures Analysis

**Figure 3:**
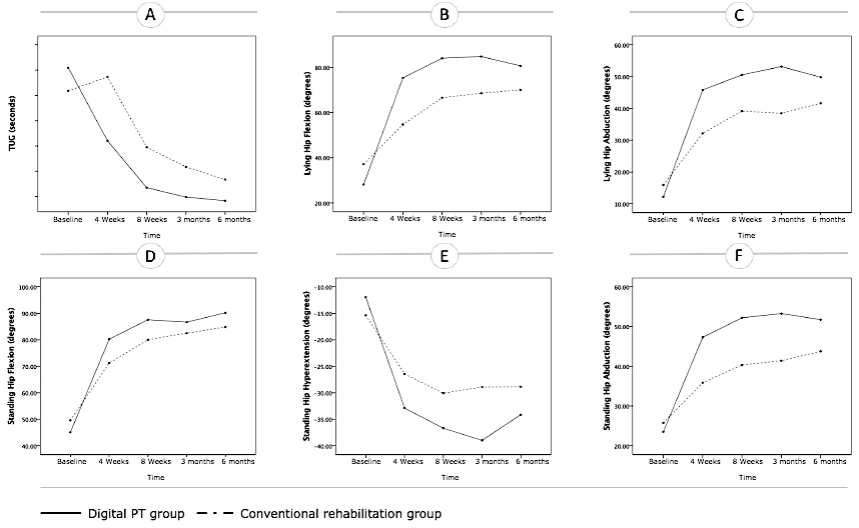
Evolution of the outcomes over time in both groups based on the repeated measures analysis (estimated marginal means are presented). (A) Timed Up and Go (TUG) score, (B) lying hip flexion, (C) lying hip abduction, (D) standing hip flexion, (E) standing hip hyperextension, (F) standing hip abduction. PT: physiotherapy.

A repeated measures ANOVA was performed only for variables with normal distribution—TUG (after log transformation) and hip ROM—and results are summarized in Table 4. Although both groups presented an improvement in every dimension evaluated, this analysis revealed a main effect of time, a main effect of group (here with the exception of the standing hip flexion ROM), and an interaction between time and group for all outcome measures in favor of the digital PT group (see Table 5 and Figure 3).

**Table 5:** Outcomes assessment: repeated measures analysis.

Outcome variable		Time		Group		Time*Group	
		*F* (df1,df2)	*P* value	*F* (df1,df2)	*P* value	*F* (df1,df2)	*P* value
**Patient performance**							
	Timed Up and Go^a,b^	128.6 (2.5,159.6)	<.001	12.3 (1,64)	.01	14.9 (3.2,159.6)	<.001
**Hip range of motion^b^**							
	Lying hip flexion	119.4 (1.9,121.6)	<.001	6.5 (1,64)	.01	12.0 (1.9,121.6)	<.001
	Lying hip abduction	139.0 (2.9,188.1)	<.001	9.4 (1,64)	.03	10.4 (2.9,121.6)	<.001
	Standing hip flexion	154.9 (1.9,123.1)	<.001	1.06 (1,64)	.31	4.0 (1.9,123.1)	.02
	Standing hip hyperextension	91.1 (3.3,211.2)	<.001	4.6 (1,64)	.04	8.2 (3.3,211.2)	<.001
	Standing hip abduction	125.5 (2.1,137.3)	<.001	10.0 (1,64)	.002	12.1 (2.1,137.3)	<.001

^a^ln transformation.

^b^Greenhouse-Geisser correction.

## Discussion

Patient refusal and consent withdrawal were the main reasons for screening failures in this study (57.7%, 90/156). The explanation for this high refusal rate resides in patient skepticism on the patient side, especially in an older population with little technological literacy. This same difficulty was reported by other authors in studies with similar devices [44] and is one of the challenges that these technologies need to overcome. The oldest patients in this study were also afraid of hidden costs, even though it was clear and thoroughly explained that participation in the study did not imply any cost.

There were two dropouts in the digital PT group, and a high percentage of patients needed assistance from a caregiver to interact with the system (37%, 13/35) or required assistance calls. This likely represents the challenges felt by an older population when dealing with technology and some issues with the user interface that need to be overcome. In particular, each physical interaction (ie, the need to calibrate sensors and the multiple touches needed to start a session) represent huge hurdles for elderly patients. This has been another challenge faced by similar technologies and is an aspect where there is still much room for improvement.

The patient satisfaction score was very high, with all but two patients rating the system with a 10/10. This is particularly interesting considering the high percentage of patients who needed assistance in using the system. When they were asked to elaborate on the reasons, almost all referred to the possibility of performing sessions at home, at their convenience. Still, it must be considered that patients who agreed to enter the study were more prone to use new technologies, and thus more likely to give high scores.

Regarding clinical outcomes, considering the reference values for the TUG [43], HOOS [45], and hip ROM [46], both groups attained clinically relevant improvements in all outcome measures in the short- and medium-term assessments. This is in line with the findings of other authors who reported the effectiveness of early exercise interventions post-THA [8,10,47-49].

Greater benefits were observed in the digital PT group, which was particularly evident in the per-protocol analysis, for all outcome measures. Furthermore, for TUG and hip ROM, these were confirmed in the repeated measures analysis. This is a major achievement for remotely assisted PT programs, considering no evidence exists yet on the superiority of a specific exercise intervention post-THA [13,50-52]. Indeed, this approach could be a game-changer on how rehabilitation programs are delivered following hip replacement. By offering a scalable solution that does not rely entirely on human resources and maximizes the reach of existing resources, while minimizing patient discomfort and the need for traveling back and forth, access to effective rehabilitation could be democratized.

A synergy of factors might explain the results obtained in this study. These have already been discussed in a previous paper [34] and can be summarized as follows: (1) beneficial impact of biofeedback and gamification on patient engagement and performance, namely on achieving a higher ROM and on a more effective correction of movement errors; (2) greater patient empowerment, coupled with the effect of monitoring on patient effort; and (3) program changes based on objective data.

In the absence of studies using technologies similar to this one, it was nearly impossible to establish interstudy comparisons. In fact, we found five reports on biofeedback systems designed to complement physical therapists’ intervention following hip arthroplasty [17,32,33,53,54], of which only two were based on inertial motion tracking [53,54]. However, the aims of these studies were distinct from ours and did not propose any rehabilitation program. Furthermore, reports on PT interventions for THA recipients revealed high methodological variability regarding timing, duration and intensity, outcome measures, and timelines for assessment [5,6,51,55]. Thus, only broad comparisons can be made between this study and previous ones.

Despite being one of the most often used and recommended performance-based outcome measures [13], the TUG test was only found in four studies [24,25,30,56]. From these, one compared the change between baseline and 9 to 12 months postsurgery [30], and the others presented data on 4- [56], 8- [24], 12- and 26-week [25] assessments or on the change between baseline and 9 to 12 months [30]. All studies but one [56] reported similar significant improvements on the TUG test with time in both intervention groups. Overall, reported changes in TUG scores varied between 0.36 seconds [56] and −5.8 seconds [25]. The results in the conventional PT group from this study fall broadly within these values, whereas the results of the digital PT group were higher, even surpassing the scores previously reported for healthy, community-living older adults (mean 8 seconds) [57,58]. Additionally, although the pattern of recovery from the conventional group followed a similar trend to the ones found in other studies using conventional PT [59,60], patients from the digital PT group improved faster (38% at 4 weeks after surgery) and to a greater extent in the medium term (60% at 24 weeks). Indeed, in the study from Naylor et al [59], an Australian cohort of 44 THA recipients (mean age 65 years) with TUG baseline values similar to ours (18 seconds), patient recovery at 4 weeks was approximately 6% and plateaued at 36% 24 weeks after surgery. Additionally, Kennedy et al [60] reported a very slow recovery in a Canadian cohort of 68 patients (mean age 68 years), with a 78% TUG aggravation within the first 4 weeks following surgery (18 seconds) and a 21% improvement from baseline after 24 weeks. However, in this latter case, baseline values were oddly low (10.14 seconds), masking an actual 73% recovery after 24 weeks when the postoperative TUG (30 seconds) was set as the reference value.

Regarding HOOS, all subscales from both groups presented higher scores than those reported on a French (N=30; 37.5-55.3 points) [45] or Swedish HOOS validation study (N=90; 56.3-82.3 points) [42] 3 and 6 months after THA, respectively. In another randomized controlled trial (RCT; N=68) on the effect of a walking skill training program in THA patients, significant improvements were detected between 3 and 5 months. However, changes were much smaller than those we observed. Also, in terms of changes from baseline, both the digital PT and the control group improved significantly from baseline to 4 weeks postoperatively, which was sooner than what was reported by Mikkelsen et al (RCT; N=73) [8] and Heiberg et al (RCT; N=68) [61]. Importantly, a ceiling effect was observed on the HOOS symptoms and pain subscales, with patients from both intervention groups reporting the best possible score from 8 weeks onward. Ceiling effects have also been reported on all subscales in the Swedish HOOS validation study, 6 months after THR [42], and in the Dutch RCT by Mikkelsen et al [8]. Considering some sensitivity is lost using this scale, a revision and adaptation to the context of digital interventions, such as the one we presented, would be very useful in the future.

Regarding hip ROM, all reports use goniometry as a means to measure hip ROM, whereas we applied high-precision sensor-based technology to assess active hip ROM, enabling continuous remote monitoring [34,62], while eliminating operator errors [63]. In a retrospective study by Davis et al (N=1383) [64], a logistic regression model yielded three levels of postsurgery hip ROM: high (115° of flexion, 25° of abduction), average (90°-114° of flexion, 16°-24° of abduction), or low (<90° of flexion, ≤15° of abduction) motion. Considering these ranges, scores from our study revealed very high abduction amplitudes in both groups at month 6 postsurgery, particularly in the digital PT group. Indeed, we found no other reports showing superior abduction results than those reported in this study [31,56,61,65,66].

On the other hand, flexion ROM values fell in the lower range reported, revealing some room for improvement. Notwithstanding, our results from the digital PT group at month 6 (median 80.7°, IQR 24.4) were comparable to the ones reported on another prospective study (N=15) [66] on THA outcomes 12 months postsurgery (flexion mean 93.3°, SD 18.7°).

Another study by Umpierres et al (RCT, N=106) [65] also reported on the improvement of hip flexion and extension ROM following THR, with an early 2-week inpatient supervised versus unsupervised intervention. Although closer to the values reported at the 4-week assessment in this study, results from the digital PT group in our study were superior to the ones reported in this RCT. Other studies were found in which flexion and extension ROMs were higher than those we reported [31,56,61]. However, even considering possible differences related to measurement methods, high baseline angles revealed that the population in these studies was not as disabled as the one in this study.

Although the improvements achieved in hip ROM are substantial, the values are still far from those reported for healthy individuals [67].

This study has several limitations that need to be acknowledged. This was a quasi-randomized study, in which patient allocation was performed according to geographical location. This implies that even if no differences were found in demographics, comorbidities, and risk factors for adverse and clinical characteristics (except for the HOOS QoL subscale), a number of factors (eg, socioeconomic) might have influenced the results. Still, almost all the patients resided in urban areas; therefore, the authors speculate that the impact of these aspects is small, but nonetheless needs to be controlled in ensuing studies.

There was a potential selection bias toward more technologically prone recipients, given the low inclusion rate. To address this, greater involvement of the clinical teams (doctors and nursing staff) in the wards is required to overcome natural patient skepticism.

The limited context of the clinical setting, which was a low-volume orthopedic hospital, may not reflect the reality of other settings. Thus, generalization of the results needs to be confirmed in larger hospitals and multicentric trials.

The study protocol depicts slight differences between the digital PT group and conventional rehabilitation group that could be confounders. First, the total active treatment time was similar between groups. However, the intensity in the digital PT group was highly variable, and unsupervised sessions in the conventional group were not taken into consideration. These aspects also need to be homogenized and controlled in future studies. Second, the exercise program was similar in both groups, with the exception of additional exercises that were possible only with a face-to-face intervention. In this sense, although the authors agree that these may be confounding factors, they benefit the conventional group and not the digital intervention group and therefore do not bias results toward the latter.

There was a notable absence of minor adverse events, in particular after 8 weeks, most likely due to underreporting. In future studies, in addition to direct telephone contacts at predetermined time stamps and specific questioning of adverse events in assessment appointments, event logs should be delivered to the patients for them to fill in.

In conclusion, this study demonstrates that home-based rehabilitation with this novel digital biofeedback system is feasible and safe following THA as previously demonstrated for TKA, and is associated with high patient satisfaction, albeit with room for improvement in terms of usability by elderly patients. Plus, to our knowledge, it is the first study demonstrating that a digital rehabilitation solution can reduce the dependence on human resources while ensuring better clinical outcomes than conventional rehabilitation in the short and medium term following THA. These promising results justify further investigation and prove the feasibility of larger RCTs to confirm these findings.

## Appendix

Multimedia Appendix 1 Rehabilitation protocol.

Multimedia Appendix 2 Raw data.

Multimedia Appendix 3 CONSORT‐EHEALTH checklist (V 1.6.1).

Multimedia Appendix 4 Intent-to-treat analysis.

Multimedia Appendix 5 Per protocol analysis.

## Abbreviations

ADL: activities of daily living
CONSORT: Consolidated Standards of Reporting Trials
HOOS: Hip dysfunction and Osteoarthritis Outcome Scale
IQR: interquartile range
ITT: intention-to-treat
PT: physiotherapy
QoL: quality of life
RCT: randomized controlled trial
ROM: range of motion
THA: total hip arthroplasty
TKA: total knee arthroplasty
TUG: Timed Up and Go
