# Supplementary material for: Digital Versus Conventional Rehabilitation After Total Hip Arthroplasty: A Single-Center, Parallel-Group Pilot Study
Source: JMIR Rehabil Assist Technol. 2019 Jun 21;6(1):e14523. doi: 10.2196/14523 (PMC6611148; doi:10.2196/14523)
Supplement: Multimedia Appendix 1 [file rehab_v6i1e14523_app1.docx]

**Rehabilitation protocols**

| **Stage 1 (weeks 0-5)** | |
| --- | --- |
| **Objectives** | **Precautions** |
| Decrease pain and swelling | Avoid hip internal rotation and hip adduction beyond neutral |
| Restore range of motion |  |
| Strengthen hip flexors and abductors | Avoid hip flexion above 90º |
| Restore fully load capacity on both legs | Ice pack application after each session and throughout the day as needed |
|  |  |
| **Intervention** | |
| **Experimental Group** | **Conventional rehabilitation** |
| Open kinetic chain exercises without added resistance | Soft tissue massage |
| Lying: | Active assisted mobilization of the hip to increase range of motion |
| - Hip flexion (2x10 reps) | Gait training with bilateral support |
| - Hip abduction (2x10 reps) | Isometric exercises |
| - Knee flexion (2x10 reps) | - gluteus contraction (3x10 sec) |
| - Hip flexion with knee flexion (2x10 reps) | - quadriceps contraction (3x10 sec) |
|  | - abductors contraction (3x10 sec) |
| Sitting (high chair): |  |
| - Hip abduction (2x10 reps) | Progressing to open kinetic chain exercises without added resistance according to patient tolerance |
| - Knee extension (2x10 reps) |  |
| - Sit to stand (2x10 reps) |  |
|  |  |
| Standing (initially with support): | - Same exercises as the experimental group |
| - Hip flexion (2x10 reps) |  |
| - Hip abduction (2x10 reps) |  |
| - Hip hyperextension (2x10 reps) |  |
| - Knee flexion (2x10 reps) |  |
| - Hip flexion with knee flexion (2x10 reps) |  |
| - Mini-squats (2x10reps) |  |
|  |  |
| **Note 1:** adjust sets, reps and total session duration according to patient tolerance (based on patient performance and on the pain and fatigue scores attributed by the patient at the end of each session) | **Note 1:** adjust sets, reps and total session duration according to patient tolerance |
|  | **Note 2:** recommend additional sessions twice per week (write down exercises, sets and reps) |
| **Note 2:** aim for at least 30 minutes in total |  |
| **Note 3:** recommend two daily sessions as soon as tolerated |  |
| **Stage 2 (weeks 6-8)** | |
| **Objectives** | **Precautions** |
| Strengthening of hip flexors and abductors | Identical to stage 1 |
| Improve balance |  |
| Independence on all activities of daily living |  |
| **Intervention** | |
| **Experimental Group** | **Conventional rehabilitation** |
| Open kinetic chain exercises in the lying, sitting and standing positions | Soft tissue massage |
|  | Balance exercises with progression to one-leg support |
| Same exercises as above but with higher number of repetitions and added resistance | Gait training with progressive withdrawal of external support |
| Progression to closed kinetic chain exercises: | Open kinetic chain exercises in the lying, sitting and standing positions |
| - Squat (2x10) |  |
| - Forward and lateral lunges (2x10) | Same exercises as above but with higher number of repetitions and added resistance |
| Stair climbing exercises |  |
| - Climb a step (2x15 steps) | Progression to closed kinetic chain exercises |
| - Come down a step (2x15 reps) |  |
|  | - same exercises as the experimental group |
| **Note 1:** adjust sets, reps and total session duration according to patient tolerance (based on patient performance and on the pain and fatigue scores attributed by the patient at the end of each session) | **Note 1:** adjust sets, reps and total session duration according to patient tolerance |
| **Note 2:** maintain recommendation of two sessions/day |  |
