# Supplementary material for: Digital Versus Conventional Rehabilitation After Total Hip Arthroplasty: A Single-Center, Parallel-Group Pilot Study
Source: JMIR Rehabil Assist Technol. 2019 Jun 21;6(1):e14523. doi: 10.2196/14523 (PMC6611148; doi:10.2196/14523)
Supplement: Multimedia Appendix 4 [file rehab_v6i1e14523_app4.docx]

Digital Versus Conventional Rehabilitation After Total Hip Arthroplasty: a Single-Center, Parallel-Group, Pilot Study

Intention-to-treat analysis

**Table 1: Primary outcome assessment: intention to treat analysis.**

| **Primary outcome - Timed up and Go*** | | | | | |
| --- | --- | --- | --- | --- | --- |
| **Time-point** | **Digital PT**  **Group**  **(n=35)** | **Control**  **Group**  **(n=31)** | ***P* value^#^** | **Estimate difference between groups** | **95% confidence**  **interval** |
| **Baseline** | 17.50 (6.3) | 14.89 (9.4) | .12 | 2.34 | -0.69;5.17 |
| **4 Weeks** | 9.92 (5.4) | 15.01 (8.2) | <.001 | -4.64 | -7.01;-2.65 |
| **Change baseline- 4 weeks** | -6.60 (8.1) | 0.53 (8.7) | <.001 | -7.90 | -10.77;-4.66 |
| **8 weeks** | 7.26 (2.1) | 11.03 (6.8) | <.001 | -3.34 | -5.14;-1.70 |
| **Change baseline-8 weeks** | - 10.50 (7.4) | - 2.90 (7.1) | <.001 | -6.33 | -8.79;-3.42 |
| **3 months** | 6.17 (2.6) | 9.63 (5.7) | <.001 | -2.50 | -4.28;-1.23 |
| **Change baseline-3 months** | -10.50 (7.7) | -4.62 (8.0) | .001 | -5.66 | -8.30;-2.36 |
| **6 months** | 6.38 (2.3) | 8.20 (4.2) | <.001 | -1.87 | -3.02;-0.62 |
| **Change baseline-6 months** | -10.50 (7.4) | -5.10 (6.9) | .005 | -4.79 | -7.24;-1.70 |

**Legend:** *Medians and IQR are presented; # Mann-Whitney U test.

**Table 2: Patient reported outcomes assessment: intention to treat analysis.**

| **Patient Reported Outcomes Assessment- HOOS*** | | | | | |
| --- | --- | --- | --- | --- | --- |
| **Variable** | **Digital PT**  **Group** | **Control**  **Group** | ***P***  **value^#^** | **Estimate difference between groups** | **95% confidence interval** |
| **Baseline** | | | | | |
| **Symptoms** | 35.0 (20.0) | 40.0 (30.0) | .12 | -10.0 | -20.0;0.0 |
| **Pain** | 33.0 (13.0) | 33.0 (35.0) | .50 | -3.0 | -13.0;5.0 |
| **Act. Daily Living** | 29.0 (15.0) | 28.0 (28.0) | .75 | 1.0 | -6.0;7.0 |
| **Sports** | 0.0 (6.0) | 0.0 (19.0) | .34 | 0.0 | 0.0;0.0 |
| **Quality of Life** | 13.0 (13.0) | 19.0 (25.0) | .03 | -6.0 | -13.0;0.0 |
| **4 weeks** | | | | | |
| **Symptoms** | 90.0 (20.0) | 90.0 (20.0) | .94 | 0.0 | -5.0;5.0 |
| **Pain** | 85.0 (35.0) | 90.0 (15.0) | .57 | -3.0 | -10.0;3.0 |
| **Act. Daily Living** | 78.0 (25.0) | 75.0 (16.0) | .59 | 1.0 | -5.0;7.0 |
| **Sports** | 19.0 (25.0) | 31.0 (19.0) | .08 | -6.0 | -13.0;0.0 |
| **Quality of Life** | 56.0 (25.0) | 50.0 (25.0) | .73 | 0.0 | -13.0;7.0 |
| **Change baseline- 4 weeks** | | | | | |
| **Symptoms** | 50.0 (30.0) | 35.0 (35.0) | .14 | 10.0 | -5.0;20.0 |
| **Pain** | 53.0 (27.0) | 43.0 (25.0) | .91 | 0.0 | -10.0; 12.0 |
| **Act. Daily Living** | 44.0 (24.0) | 35.0 (27.0) | .55 | 3.0 | -9.0;12.0 |
| **Sports** | 19.0 (32.0) | 25.0 (18.0) | .43 | -6.0 | -12.0;6.0 |
| **Quality of Life** | 38.0 (31.0) | 31.0 (31.0) | .41 | 6.0 | -6.0;13.0 |
| **8 weeks** | | | | | |
| **Symptoms** | 100.0 (5.0) | 95.0 (20.0) | .01 | 5.00 | 0.0;10.0 |
| **Pain** | 100.0 (7.0) | 98.0 (12.0) | .24 | 0.0 | 0.0; 5.0 |
| **Act. Daily Living** | 93.0 (11.0) | 82.0 (14.0) | <.001 | 9.0 | 4.0;13.0 |
| **Sports** | 50.0 (18.0) | 38.0 (19.0) | .004 | 12.0 | 6.0;19.0 |
| **Quality of Life** | 81.0 (19.0) | 69.0 (31.0) | .08 | 6.0 | 0.0;18.0 |
| **Change baseline- 8 weeks** | | | | | |
| **Symptoms** | 60.0 (30.0) | 45.0 (30.0) | .06 | 10.0 | 0.0;20.0 |
| **Pain** | 60.0 (22.0) | 60.0 (32.0) | .75 | 2.0 | -10.0;10.0 |
| **Act. Daily Living** | 56.0 (23.0) | 57.0 (27.0) | .63 | -2.0 | -10.0;6.0 |
| **Sports** | 44.0 (25.0) | 38.0 (25.0) | .26 | 6.0 | -6.0;13.0 |
| **Quality of Life** | 63.0 (31.0) | 50.0 (25.0) | .46 | 6.0 | -6.0;13.0 |
| **3 months** | | | | | |
| **Symptoms** | 100.0 (5.0) | 95.0 (15.0) | .08 | 5.00 | 0.0;10.0 |
| **Pain** | 100.0 (5.0) | 98.0 (12.0) | 0.10 | 0.0 | 0.0; 2.0 |
| **Act. Daily Living** | 94.0 (10.0) | 87.0 (21.0) | <.001 | 7.0 | 3.0;12.0 |
| **Sports** | 56.0 (19.0) | 44.0 (25.0) | .03 | 7.0 | 0.0;19.0 |
| **Quality of Life** | 81.0 (13.0) | 75.0 (21.0) | .004 | 12.0 | 6.0;19.0 |
| **Change baseline-3 months** | | | | | |
| **Symptoms** | 60.0 (25.0) | 50.0 (35.0) | .04 | 15.0 | 0.0;25.0 |
| **Pain** | 65.0 (22.0) | 53.0 (33.0) | .16 | 10.0 | -3.0;20.0 |
| **Act. Daily Living** | 65.0 (16.0) | 50.0 (25.0) | .02 | 10.0 | 2.0;18.0 |
| **Sports** | 50.0 (19.0) | 38.0 (25.0) | .01 | 13.0 | 6.0;19.0 |
| **Quality of Life** | 69.0 (19.0) | 50.0 (25.0) | <.001 | 19.0 | 12.0;31.0 |
| **6 months** | | | | | |
| **Symptoms** | 100.0 (5.0) | 95.0 (10.0) | .20 | 0.0 | 0.0;5.0 |
| **Pain** | 100.0 (5.0) | 100.0 (7.0) | .75 | 0.0 | 0.0;0.0 |
| **Act. Daily Living** | 96.0 (11.0) | 88.0 (19.0) | .02 | 4.0 | 0.0;10.0 |
| **Sports** | 75.0 (32.0) | 50.00 (32.0) | .01 | 19.0 | 6.0;37.0 |
| **Quality of Life** | 94.0 (12.0) | 81.0 (19.0) | .02 | 7.0 | 0.0;19.0 |
| **Change baseline-6 months** | | | | | |
| **Symptoms** | 60.0 (25.0) | 45.0 (30.0) | .06 | 10.0 | 0.0;20.0 |
| **Pain** | 65.0 (18.0) | 53.0 (30.0) | .21 | 7.0 | -5.0;17.0 |
| **Act. Daily Living** | 63.0 (22.0) | 56.0 (25.0) | .10 | 7.0 | -1.0;15.0 |
| **Sports** | 69.0 (31.0) | 38.0 (38.0) | .004 | 25.0 | 7.0;37.0 |
| **Quality of Life** | 75.0 (32.0) | 56.0 (31.0) | .01 | 19.0 | 6.0;25.0 |

**Legend:** *Medians and IQR are presented; # Mann-Whitney U test.

**Table 3: Hip range of motion outcomes assessment: intention to treat analysis.**

| **Hip range of motion assessment*** | | | | | |
| --- | --- | --- | --- | --- | --- |
| **Variable** | **Digital PT**  **Group** | **Control**  **Group** | ***P***  **value^#^** | **Estimate difference between groups** | **95% confidence interval** |
| **Baseline** | | | | | |
| **Lying Flexion** | 28.2 (19.1) | 37.1 (20.0) | .07 | -8.9 | -18.53;0.67 |
| **Lying Abduction** | 12.2 (5.4) | 15.9 (9.1) | .05 | -3.7 | -7.48;0.02 |
| **Standing Flexion** | 45.1 (15.9) | 49.6 (16.7) | .27 | -4.5 | -12.52;3.53 |
| **Standing Hyperext** | -11.9 (7.0) | -15.4 (8.8) | .31 | 3.4 | -0.44;7.33 |
| **Standing Abduction** | 23.5 (6.8) | 25.8 (10.7) | .08 | -2.2 | -6.78;2.26 |
| **4 weeks** | | | | | |
| **Lying Flexion** | 75.3 (26.7) | 54.7 (21.3) | .001 | 20.6 | 8.67;32.6 |
| **Lying Abduction** | 45.8 (16.6) | 32.2 (10.2) | <.001 | 13.6 | 6.75;20.53 |
| **Standing Flexion** | 80.2 (19.9) | 71.2 (17.0) | .054 | 9.0 | -0.14;18.15 |
| **Standing Hyperext** | -32.9 (13.5) | -26.5 (7.5) | .02 | -6.4 | -11.73;-1.13 |
| **Standing Abduction** | 47.3 (12.8) | 35.9 (10.8) | <.001 | 11.4 | 5.55:17.28 |
| **Change baseline-4 weeks** | | | | | |
| **Lying Flexion** | 47.1 (29.5) | 17.5 (27.7) | <.001 | 29.6 | 15.45;43.74 |
| **Lying Abduction** | 33.6 (15.8) | 16.3 (12.3) | <.001 | 17.4 | 10.36;24.38 |
| **Standing Flexion** | 35.1 (18.7) | 21.6 (20.0) | .01 | 13.5 | 3.95;22.99 |
| **Standing Hyperext** | -20.9 (11.6) | -11.1 (9.1) | <.001 | -9.8 | -15.03;-4.66 |
| **Standing Abduction** | 23.8 (12.2) | 10.1 (12.8) | <.001 | 13.7 | 7.51;19.90 |
| **8 weeks** | | | | | |
| **Lying Flexion** | 84.0 (23.5) | 66.6 (19.6) | .002 | 17.5 | 6.78;28.18 |
| **Lying Abduction** | 50.5 (17.5) | 39.2 (15.2) | .01 | 11.4 | 3.27:19.50 |
| **Standing Flexion** | 87.6 (21.2) | 80.0 (19.8) | .14 | 7.5 | -2.58;17.66 |
| **Standing Hyperext** | -36.7 (14.3) | -30.1 (8.2) | .03 | -6.6 | -12.28;-0.96 |
| **Standing Abduction** | 52.2 (13.8) | 40.3 (11.3) | <.001 | 11.9 | 5.62;18.13 |
| **Change baseline- 8 weeks** | | | | | |
| **Lying Flexion** | 55.8 (27.4) | 29.4 (25.6) | <.001 | 26.4 | 13.32;39.50 |
| **Lying Abduction** | 38.4 (17.3) | 23.3 (15.7) | <.001 | 15.1 | 6.91;23.25 |
| **Standing Flexion** | 42.5 (21.3) | 30.4 (20.3) | .02 | 12.0 | 1.81;22.33 |
| **Standing Hyperext** | -24.7 (12.7) | -14.7 (10.1) | .001 | -10.1 | -15.75;-4.38 |
| **Standing Abduction** | 28.7 (13.4) | 14.6 (13.5) | <.001 | 14.1 | 7.51;20.76 |
| **3 months** | | | | | |
| **Lying Flexion** | 84.8 (22.0) | 68.6 (19.3) | .002 | 16.2 | 5.93;26.44 |
| **Lying Abduction** | 53.1 (16.9) | 38.5 (13.8) | <.001 | 14.7 | 7.03;22.36 |
| **Standing Flexion** | 86.7 (22.1) | 82.5 (17.7) | .41 | 4.1 | -5.79;14.07 |
| **Standing Hyperext** | -39.0 (15.1) | -28.9 (10.0) | .002 | -10.1 | -16.45;3.68 |
| **Standing Abduction** | 53.3 (14.3) | 41.4 (12.1) | .001 | 11.8 | 5.27;18.40 |
| **Change baseline-3 months** | | | | | |
| **Lying Flexion** | 56.6 (27.0) | 31.5 (25.2) | <.001 | 25.1 | 12.24;38.00 |
| **Lying Abduction** | 41.0 (16.8) | 22.6 (15.0) | <.001 | 18.4 | 10.52;26.26 |
| **Standing Flexion** | 41.5 (23.1) | 32.9 (18.9) | .11 | 8.6 | -1.87;19.08 |
| **Standing Hyperext** | -27.0 (13.8) | - 13.5 (10.2) | <.001 | -13.5 | -19.52;-7.43 |
| **Standing Abduction** | 29.7 (14.1) | 15.6 (13.4) | <.001 | 14.1 | 7.32;20.88 |
| **6 months** | | | | | |
| **Lying Flexion** | 80.7 (24.4) | 70.0 (19.3) | .06 | 10.7 | -0.27;21.6 |
| **Lying Abduction** | 49.8 (18.2) | 41.6 (14.3) | .048 | 8.2 | 0.06;16.31 |
| **Standing Flexion** | 90.2 (23. 1) | 84.8 (19.8) | .32 | 5.4 | -5.25; 16.03 |
| **Standing Hyperext** | -34.1 (15.1) | -28.8 (9.2) | .10 | -5.3 | -11.36; 0.81 |
| **Standing Abduction** | 51.7 (15.1) | 43.8 (11.8) | .02 | 8.0 | 1.24;14.69 |
| **Change baseline-6 months** | | | | | |
| **Lying Flexion** | 52.5 (26.6) | 32.8 (25.6) | .003 | 19.6 | 6.73;32.50 |
| **Lying Abduction** | 37.6 (18.2) | 25.7 (15.2) | .01 | 11.9 | 3.57;20.20 |
| **Standing Flexion** | 45.1 (22.6) | 35.2 (20.6) | .07 | 9.9 | -0.79;20.57 |
| **Standing Hyperext** | -22.2 (13.3) | -13.5 (11.1) | .01 | -8.7 | -14.72;-2.59 |
| **Standing Abduction** | 28.2 (14.3) | 18.0 (12.1) | .003 | 10.2 | 3.64;16.74 |

**Legend:** *medians and IQR are presented; # Mann-Whitney U test.
