# Supplementary material for: Digital Versus Conventional Rehabilitation After Total Hip Arthroplasty: A Single-Center, Parallel-Group Pilot Study
Source: JMIR Rehabil Assist Technol. 2019 Jun 21;6(1):e14523. doi: 10.2196/14523 (PMC6611148; doi:10.2196/14523)
Supplement: Multimedia Appendix 5 [file rehab_v6i1e14523_app5.docx]

Digital Versus Conventional Rehabilitation After Total Hip Arthroplasty: a Single-Center, Parallel-Group, Pilot Study

Per protocol analysis

**Table 1. Primary outcome assessment: per protocol analysis.**

| **Primary outcome - Timed up and Go*** | | | | | |
| --- | --- | --- | --- | --- | --- |
| **Time-point** | **Digital PT Group**  **(n=30)** | **Control**  **Group**  **(n=28)** | ***P* value^#^** | **Estimate difference between groups** | **95% confidence**  **interval** |
| **Baseline** | 17.55 (7.0) | 14.89 (9.1) | .11 | 2.55 | -0.64; 5.73 |
| **4 Weeks** | 9.73 (5.1) | 15.01 (7.4) | <.001 | -4.89 | -7.10;-2.84 |
| **Change baseline- 4 weeks** | -6.87 (7.6) | 0.70 (9.7) | <.001 | -8.50 | -11.57;-4.89 |
| **8 weeks** | 6.85 (2.0) | 11.03 (6.3) | <.001 | -3.53 | -5.38;-1.93 |
| **Change baseline-8 weeks** | - 10.79 (5.0) | - 3.63 (7.0) | <.001 | -6.87 | -9.23;-4.05 |
| **3 months** | 5.87 (1.7) | 8.90 (5.4) | <.001 | -2.74 | -4.52;-1.49 |
| **Change baseline-3 months** | -11.72 (5.9) | -4.72 (7.8) | <.001 | -6.16 | -8.85;-3.04 |
| **6 months** | 5.96 (1.8) | 7.83 (3.8) | <.001 | -2.04 | -3.20;-1.09 |
| **Change baseline-6 months** | -11.15 (5.7) | -5.67 (7.1) | .001 | -5.22 | -7.71;-2.38 |

**Legend:** *Medians and IQR are presented; # Mann-Whitney U test

**Table 2. Patient reported outcomes assessment: per protocol analysis.**

| **Patient Reported Outcomes Assessment- HOOS*** | | | | | |
| --- | --- | --- | --- | --- | --- |
| **Variable** | **Digital PT Group** | **Control**  **Group** | ***P***  **value^#^** | **Estimate difference between groups** | **95% confidence interval** |
| **Baseline** | | | | | |
| **Symptoms** | 35.0 (30.0) | 45.0 (33.0) | .13* | 10.0 | 0.0;20.0 |
| **Pain** | 33.0 (14.0) | 33.0 (37.0) | .35* | 5.0 | -5.0;15.0 |
| **Act. Daily Living** | 28.5 (15.0) | 38.0 (28.0) | .95* | 0.0 | -7.0;6.0 |
| **Sports** | 0.0 (6.0) | 5.0 (16.0) | .38* | 0.0 | 0.0;0.0 |
| **Quality of Life** | 13.0 (19.0) | 19.0 (22.0) | .07* | 6.0 | 0.0;13.0 |
| **4 weeks** | | | | | |
| **Symptoms** | 90.0 (16.0) | 90.0 (18.0) | .39 | 0.0 | -5.0;10.0 |
| **Pain** | 85.0 (28.0) | 90.0 (15.0) | .7 | 0.0 | 0.0; 5.0 |
| **Act. Daily Living** | 78.0 (18.0) | 75.0 (14.0) | .45 | 3.0 | -3.0;9.0 |
| **Sports** | 19.0 (25.0) | 31.0 (19.0) | .09 | 6.0 | 0.0;13.0 |
| **Quality of Life** | 56.0 (25.0) | 51.0 (25.0) | .99 | 0.0 | -7.0;12.0 |
| **Change baseline- 4 weeks** | | | | | |
| **Symptoms** | 55.0 (26.3) | 35.0 (32.5) | .02 | 15.0 | 5.0;25.0 |
| **Pain** | 52.0 (25.5) | 43.0 (26.0) | .48 | 3.0 | -8.0; 13.0 |
| **Act. Daily Living** | 44.0 (22.3) | 35.0 (25.0) | .26 | 4.0 | -5.0;13.0 |
| **Sports** | 19.00 (26.8) | 25.0 (15.0) | .48 | 6.0 | -7.0;12.0 |
| **Quality of Life** | 38.0 (26.5) | 31.0 (28.5) | .26 | 6.0 | 6.0;13.0 |
| **8 weeks** | | | | | |
| **Symptoms** | 100.0 (5.0) | 95.0 (17.5) | <.001* | 5.00 | 5.0;15.0 |
| **Pain** | 100.0 (7.0) | 98.0 (11.0) | .08* | 0.0 | 0.0; 5.0 |
| **Act. Daily Living** | 93.5 (8.3) | 82.0 (13.5) | <.001* | 9.0 | 6.0;14.0 |
| **Sports** | 56.0 (13.8) | 38.0 (19.0) | <.001* | 13.0 | 6.0;19.0 |
| **Quality of Life** | 81.0 (19.0) | 69.0 (31.0) | .013* | 12.0 | 0.0;19.0 |
| **Change baseline- 8 weeks** | | | | | |
| **Symptoms** | 65.0 (25.0) | 45.0 (28.0) | <.001* | 20.0 | 1.0;30.0 |
| **Pain** | 65.0 (16.0) | 48.0 (27.0) | .03* | 12.0 | 0.0;20.0 |
| **Act. Daily Living** | 64.5 (17.0) | 50.0 (28.0) | .002* | 12.0 | 4.0;19.0 |
| **Sports** | 50.0 (18.0) | 31.0 (19.0) | <.001* | 18.0 | 7.0;25.0 |
| **Quality of Life** | 63.0 (19.0) | 44.0 (18.0) | <.001* | 19.0 | 12.0;25.0 |
| **3 months** | | | | | |
| **Symptoms** | 100.0 (0.0) | 95.0 (15.0) | <.001 | 5.00 | 0.0;10.0 |
| **Pain** | 100.00 (1.0) | 98.00 (12.0) | .02 | 0 | 0.0; 2.0 |
| **Act. Daily Living** | 96.00 (8.0) | 87.00 (11.0) | <.001 | 8.0 | 4.0;12.0 |
| **Sports** | 56.00 (22.0) | 50.00 (18.0) | .01 | 12.0 | 6.0;19.0 |
| **Quality of Life** | 78.00 (13.0) | 56.00 (38.0) | <.001 | 13.0 | 6.0;25.0 |
| **Change baseline-3 months** | | | | | |
| **Symptoms** | 65.0 (25.0) | 50.0 (35.0) | .003 | 15.0 | 5.0;30.0 |
| **Pain** | 68.0 (17.0) | 53.0 (27.0) | .02 | 13.0 | 3.0;23.0 |
| **Act. Daily Living** | 65.5 (13.2) | 51.0 (25.0) | .002 | 12.0 | 5.0;19.0 |
| **Sports** | 53.0 (20.5) | 38.0 (19.0) | .003 | 13.0 | 6.0;25.0 |
| **Quality of Life** | 69.0 (13.5) | 44.0 (32.0) | <.001 | 25.0 | 18.0;37.0 |
| **6 months** | | | | | |
| **Symptoms** | 100.0 (1.0) | 95.0 (10.0) | .02 | 0.0 | 0.0;10.0 |
| **Pain** | 100.00 (2.0) | 100.00 (7.0) | .16 | 0.0 | 0.0;2.0 |
| **Act. Daily Living** | 97.00 (5.0) | 91.00 (15.0) | .003 | 5.0 | 2.0;11.0 |
| **Sports** | 84.50 (22.0) | 75.00 (44.0) | .002 | 25.0 | 12.0;38.0 |
| **Quality of Life** | 100.00 (8.0) | 88.00 (25.0) | <.001 | 12.0 | 0.0;19.0 |
| **Change baseline-6 months** | | | | | |
| **Symptoms** | 62.5 (21.3) | 45.0 (30.0) | .004 | 15.0 | 5.0;25.0 |
| **Pain** | 66.5 (17.0) | 53.0 (27.0) | .03 | 12.0 | 0.0;20.0 |
| **Act. Daily Living** | 66.0 (16.5) | 56.0 (17.0) | .03 | 8.0 | 0.0;16.0 |
| **Sports** | 75.0 (25.0) | 44.0 (38.0) | .001 | 25.0 | 12.0;38.0 |
| **Quality of Life** | 81.0 (20.5) | 56.0 (31.0) | .001 | 19.0 | 7.0;31.0 |

**Legend:** *Medians and IQR are presented; # Mann-Whitney U test.

**Table 3. Hip range of motion outcomes assessment: per protocol analysis.**

| **Hip range of motion assessment*** | | | | | |
| --- | --- | --- | --- | --- | --- |
| **Variable** | **Digital PT Group** | **Control**  **group** | ***P***  **value^#^** | **Estimate difference between groups** | **95% confidence interval** |
| **Baseline** | | | | | |
| **Lying Flexion** | 28.9 (20.3) | 37.7 (19.0) | .09 | 8.8 | -1.4;19.1 |
| **Lying Abduction** | 12.4 (5.4) | 15.9 (8.8) | .08 | 3.4 | -0.4;7.2 |
| **Standing Flexion** | 44.9 (17.0) | 50.8 (16.7) | .18 | 5.9 | -2.9;-14.7 |
| **Standing Hyperext** | -12.4 (7.2) | -15.6 (9.1) | .14 | 2.1 | -2.6;6.9 |
| **Standing Abduction** | 24.1 (7.1) | 26.2 (10.8) | .37 | 3.2 | -7.4;1.1 |
| **4 weeks** | | | | | |
| **Lying Flexion** | 79.0 (24.1) | 57.1 (19.1) | <.001 | 5.67 | 10.5;33.2 |
| **Lying Abduction** | 48.8 (13.4) | 33.0 (9.0) | <.001 | 15.8 | 9.8;21.7 |
| **Standing Flexion** | 82.8 (17.9) | 72.9 (15.6) | .03 | 9.9 | 1.1;18.7 |
| **Standing Hyperext** | -35.6 (11.4) | -26.5 (7.1) | <.001 | 9.0 | 4.1;13.9 |
| **Standing Abduction** | 49.8 (10.4) | 36.8 (10.3) | <.001 | 13.0 | 7.6;18.4 |
| **Change baseline-4 weeks** | | | | | |
| **Lying Flexion** | 50.1 (27.0) | 19.4 (27.7) | <.001 | 30.7 | 16.4;45.0 |
| **Lying Abduction** | 36.4 (12.3) | 17.2 (12.2) | <.001 | 19.2 | 12.8;25.6 |
| **Standing Flexion** | 38.0 (16.4) | 22.1 (20.2) | .002 | 15.8 | 6.2;25.4 |
| **Standing Hyperext** | -23.2 (10.3) | -11.0 (8.9) | <.001 | -12.2 | 7.2;17.2 |
| **Standing Abduction** | 25.7 (10.3) | 10.5 (13.1) | <.001 | 15.2 | 9.0;21.3 |
| **8 weeks** | | | | | |
| **Lying Flexion** | 89.2 (17.4) | 69.8 (14.6) | <.001 | 19.4 | 11.0;27.7 |
| **Lying Abduction** | 54.3 (13.2) | 40.5 (14.2) | <.001 | 13.8 | 6.7;21.0 |
| **Standing Flexion** | 91.4 (18.1) | 82.3 (17.8) | .06 | 9.1 | -0.3;18.4 |
| **Standing Hyperext** | -40.0 (11.0) | -30.4 (7.8) | <.001 | 14.0 | 8.7;19.3 |
| **Standing Abduction** | 55.6 (10.2) | 41.6 (10.3) | <.001 | 9.6 | 4.6;14.6 |
| **Change baseline- 8 weeks** | | | | | |
| **Lying Flexion** | 60.3 (22.6) | 32.7 (24.2) | <.001 | 28.2 | 16.0;40.4 |
| **Lying Abduction** | 41.9 (13.2) | 24.7 (15.3) | <.001 | 17.2 | 9.8;24.6 |
| **Standing Flexion** | 46.5 (18.0) | 31.5 (20.2) | .004 | 15.0 | 5.0;25.0 |
| **Standing Hyperext** | - 27.7 (10.6) | -14.9 (9.9) | <.001 | 16.2 | 9.8;22.5 |
| **Standing Abduction** | 31.5 (10.7) | 15.3 (13.6) | <.001 | 12.8 | 7.4;18.2 |
| **3 months** | | | | | |
| **Lying Flexion** | 90.0 (14.70) | 72.0 (13.74) | <.001 | 18.0 | 10.6;25.5 |
| **Lying Abduction** | 57.4 (11.30) | 39.8 (12.68) | <.001 | 17.6 | 11.3;23.9 |
| **Standing Flexion** | 90.3 (19.49) | 85.0 (14.86) | .24 | 5.3 | -3.7;14.4 |
| **Standing Hyperext** | -42.7 (11.48) | -29.2 (9.81) | <.001 | -13.5 | -19.1;-7.9 |
| **Standing Abduction** | 56.8 (10.66) | 42.7 (11.05) | <.001 | 14.1 | 8.4;19.7 |
| **Change baseline-3 months** | | | | | |
| **Lying Flexion** | 61.1 (21.8) | 36.6 (22.4) | .001 | 24.6 | 12.8;36.3 |
| **Lying Abduction** | 44.9 (11.4) | 23.4 (14.1) | <.001 | 21.5 | 14.7;28.3 |
| **Standing Flexion** | 45.4 (20.7) | 35.0 (19.0) | .052 | 10.5 | -0.1;21.1 |
| **Standing Hyperext** | -30.3 (11.4) | - 13.6 (10.1) | <.001 | -16.8 | -22.5;-11.0 |
| **Standing Abduction** | 32.7 (11.4) | 16.4 (13.8) | <.001 | 16.3 | 9.6;23.0 |
| **6 months** | | | | | |
| **Lying Flexion** | 85.2 (19.88) | 74.1 (13.53) | .02 | 11.1 | 1.9;20.2 |
| **Lying Abduction** | 53.5 (14.61) | 43.2 (12.06) | .06 | 10.3 | 3.09-17.40 |
| **Standing Flexion** | 94.5 (20.01) | 88.3 (16.89) | .21 | 6.2 | -3.7; 16.1 |
| **Standing Hyperext** | -37.0 (12.98) | -29.3 (9.21) | .01 | -7.7 | -13.8; -1.7 |
| **Standing Abduction** | 55.0 (12.29) | 45.5 (10.43) | .03 | 9.5 | 3.5;15.6 |
| **Change baseline-6 months** | | | | | |
| **Lying Flexion** | 56.3 (22.3) | 38.2 (22.5) | .003 | 18.2 | 6.3;30.1 |
| **Lying Abduction** | 41.0 (14.8) | 27.1 (14.0) | .001 | 14.0 | 6.3;21.6 |
| **Standing Flexion** | 49.6 (19.2) | 37.6 (20.6) | .03 | 12.0 | 1.5;22.6 |
| **Standing Hyperext** | -24.7 (12.1) | -13.5 (11.2) | .001 | -11.1 | -17.4;-4.9 |
| **Standing Abduction** | 30.9 (12.1) | 19.1 (12.1) | .001 | 3.2 | 5.4;18.2 |

**Legend:** *Medians and IQR are presented; # Mann-Whitney U test.

**Table 4. Outcomes assessment - Repeated measures analysis according to a per protocol analysis.** ^#^ln transformation; ^&^Greenhouse-Geisser correction

| **Outcome variable** | **Time** | | **Group** | | **Time*Group** | |
| --- | --- | --- | --- | --- | --- | --- |
|  | **F(df1,df2)** | ***P*** | **F(df1,df2)** | ***P*** | **F(df1,df2)** | ***P*** |
| **Patient performance** | | | | | | |
| **TUG^#&^** | F(2.6,144.1)=150.4 | <.001 | F(1,55)=17.8 | <.001 | F(2.6,144.1)=18.0 | <.001 |
| **Hip range of motion** | | | | | | |
| **Lying Hip Flexion**^&^ | F(2.2,119.2)=147.8 | <.001 | F(1,55)=11.2 | .001 | F(2.2,119.2)=11.6 | <.001 |
| **Lying Hip Abduction**^&^ | F(3.56,195.9)=158.2 | <.001 | F(1,55)=21.3 | <.001 | F(3.5,195.9)=13.4 | <.001 |
| **Standing Hip Flexion**^&^ | F(2.2,120.2)=169.5 | <.001 | F(1,55)=1.4 | .25 | F(2.2,120.2)=5.1 | .01 |
| **Standing Hip Hyperextension**^&^ | F(3.6,200.3)=94.1 | <.001 | F(1,55)=12.2 | .001 | F(3.6,200.3)=11.3 | <.001 |
| **Standing Hip Abduction**^&^ | F(2.5,136.1)=138.5 | <.001 | F(1,55)=18.6 | <.001 | F(2.5,136.1)=15.0 | <.001 |

**
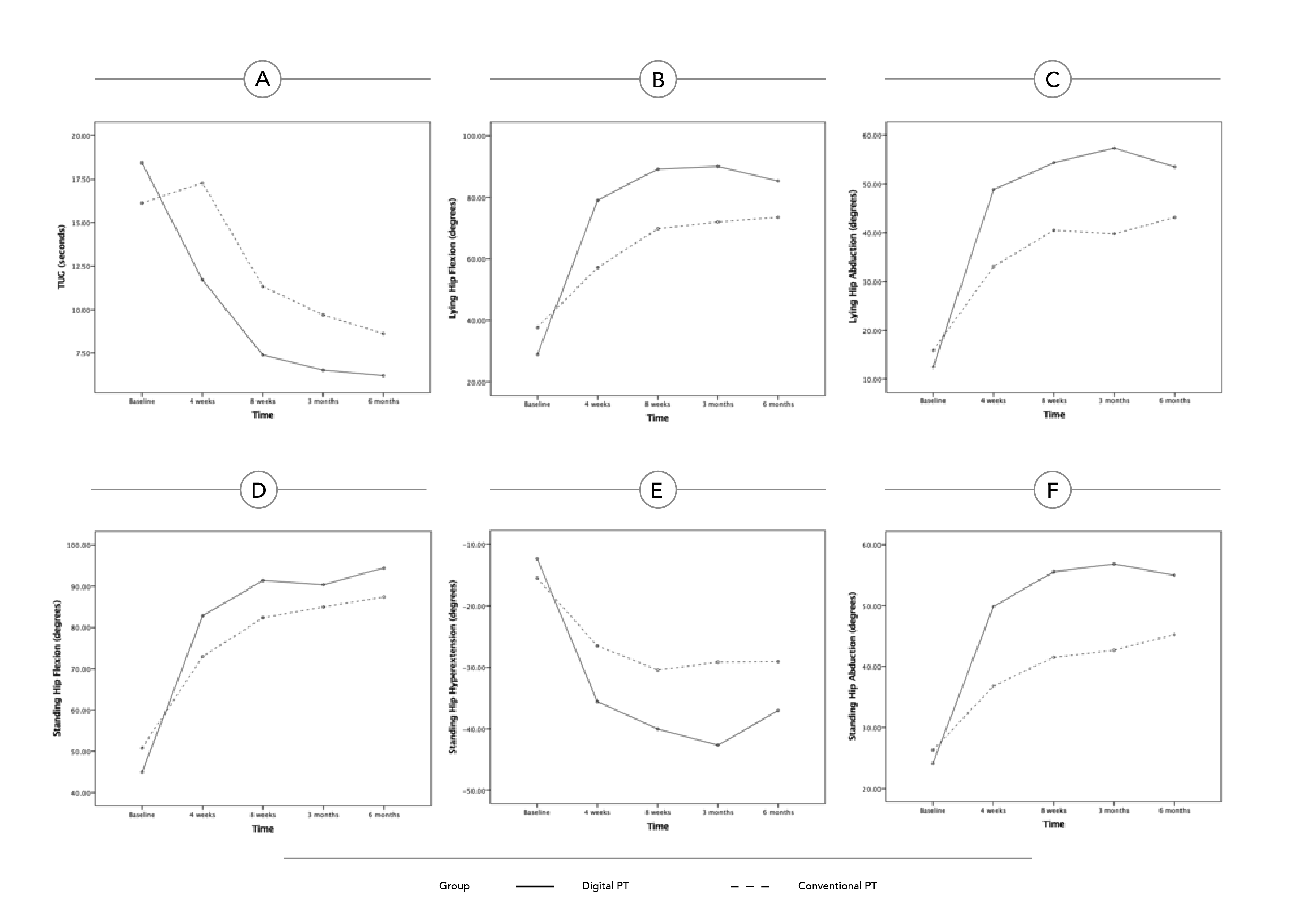
**

**Figure 1.** Evolution of outcomes over time in the THA study: per protocol analysis (estimated marginal means are presented). A- TUG score; B- Lying hip flexion; C- Lying hip abduction; D- Standing hip flexion; E- Standing hip hyperextension; F-Standing hip abduction. PT: physiotherapy.
